# A longitudinal and geospatial analysis of COVID-19 tweets during the early outbreak period in the United States

**DOI:** 10.1186/s12889-021-10827-4

**Published:** 2021-04-24

**Authors:** Raphael E. Cuomo, Vidya Purushothaman, Jiawei Li, Mingxiang Cai, Tim K. Mackey

**Affiliations:** 1 Department of Anesthesiology, University of California, San Diego School of Medicine, San Diego, CA USA; 2 Global Health Policy and Data Institute, San Diego, CA USA; 3 S-3 Research LLC, San Diego, CA USA; 4 Global Health Program, Department of Anthropology, University of California, San Diego, USA

**Keywords:** Ecological epidemiology, COVID-19, Infectious diseases, Geospatial analysis, Social media

## Abstract

**Introduction:**

Early reports of COVID-19 cases and deaths may not accurately convey community-level concern about the pandemic during early stages, particularly in the United States where testing capacity was initially limited. Social media interaction may elucidate public reaction and communication dynamics about COVID-19 in this critical period, during which communities may have formulated initial conceptions about the perceived severity of the pandemic.

**Methods:**

Tweets were collected from the Twitter public API stream filtered for keywords related to COVID-19. Using a pre-existing training set, a support vector machine (SVM) classifier was used to obtain a larger set of geocoded tweets with characteristics of user self-reporting COVID-19 symptoms, concerns, and experiences. We then assessed the longitudinal relationship between identified tweets and the number of officially reported COVID-19 cases using linear and exponential regression at the U.S. county level. Changes in tweets that included geospatial clustering were also assessed for the top five most populous U.S. cities.

**Results:**

From an initial dataset of 60 million tweets, we analyzed 459,937 tweets that contained COVID-19-related keywords that were also geolocated to U.S. counties. We observed an increasing number of tweets throughout the study period, although there was variation between city centers and residential areas. Tweets identified as COVID-19 symptoms or concerns appeared to be more predictive of active COVID-19 cases as temporal distance increased.

**Conclusion:**

Results from this study suggest that social media communication dynamics during the early stages of a global pandemic may exhibit a number of geospatial-specific variations among different communities and that targeted pandemic communication is warranted. User engagement on COVID-19 topics may also be predictive of future confirmed case counts, though further studies to validate these findings are needed.

## Background

In mid-March 2020, approximately 150,000 cases of coronavirus 2019 (COVID-19) had been confirmed globally, with only about 2000 of these cases occurring at the time in the United States [1]. Domestic attention to the potential threat of the COVID-19 pandemic was likely widespread [2–4], though current data do not allow for a highly valid means of estimating the extent of public concern at this earlier stage of the pandemic. Furthermore, many who became infected did not exhibit symptoms (i.e., asymptomatic cases) or exhibited very mild symptoms, complicating the relationship between reporting of case counts and actual public attention and concern to what would eventually become a global public health crisis, which as of March 2021, has claimed the lives of more than half a million Americans [5, 6].

In the first half of March 2020, limited availability of tests for COVID-19 led public health officials to suggest that only certain individuals need to seek confirmation of COVID-19 infection with a diagnostic test [7]. As a result, it was recommended that testing be reserved for individuals suffering relatively severe symptoms requiring hospitalization [8]. This may have resulted in an early outbreak period that exhibited inaccurate spatial variation for pandemic-related concern due to underreporting of true case count estimations. Hence, limited testing capacity and data on actual case estimations necessitates examination of non-traditional sources of surveillance data, including forms of syndromic and infodemiology approaches that use data generated outside of clinical settings. One approach to assessing underreporting of COVID-19 symptomatic individuals and possible cases is by using “infoveillance” approaches, including using Internet and social media data to identify the distribution and determinants of disease-related concern, such as self-reporting of COVID-19 symptoms and lack of access to testing [9–11].

A number of studies have used social media data to identify and characterize user experiences with COVID-19, including the detection of self-reporting of COVID-19 symptoms, user sentiment, information spreading, exposure to misinformation, illegal sale of COVID-19 health products, and other topics [12–16]. In addition to social media-based infoveillance, analysis of geographic distributions of online COVID-19 communication during this period may be helpful in understanding the variability in how communities interact with the topic of a novel and emerging infectious disease outbreak [17, 18]. These geographic distributions may also be valuable to public health practitioners seeking to disseminate information about preventive behaviors (e.g. mask wearing, social distancing) in the context of pandemic response, as well as to public health practitioners seeking to understand the latent capability of communities to receive and respond to pandemic-specific public health communication strategies [19–21].

Building on this prior research, this study aims to explore the use of geospatial, statistical, and machine learning methods to better understand how social media data from the popular microblogging platform Twitter can be leveraged to estimate geographic distributions of public attention and concern to the COVID-19 pandemic. Specifically, the use of Twitter data has a number of advantages in achieving the objective of understanding geospatial variability in communication during disease outbreaks. Practically, it is more feasible to obtain large volumes of unprompted and self-reported conversations closer to real-time that more immediately convey the experiences of online users compared to traditional surveys that may be retrospective and subject to recall bias [22]. The near real-time data generated by social media is particularly important when exploring changing trends in public health emergencies, such as a global pandemic. Also, the unprompted nature of these messages may encourage more organic spatiotemporal reflection of attention to a given topic, which is further facilitated by a large number of users (Twitter reports that it had 187 million active users worldwide as of January 2021) and methods to opt-in to geolocation [23, 24].

We specifically choose to examine the early outbreak period in the United States as it represents a critical time frame of formation of public perception, knowledge dissemination, and initial behavior adaptation towards public health interventions, which can influence subsequent behaviors and attitudes towards the pandemic in later stages [25]. Other studies have similarly examined other social media platforms (e.g., Chinese microblogging platform Weibo) at the early stages of COVID-19 in other countries for public perception and sentiment, such as in Wuhan City, China, where the outbreak originated [26–28].

## Methods

**Fig. 1:**
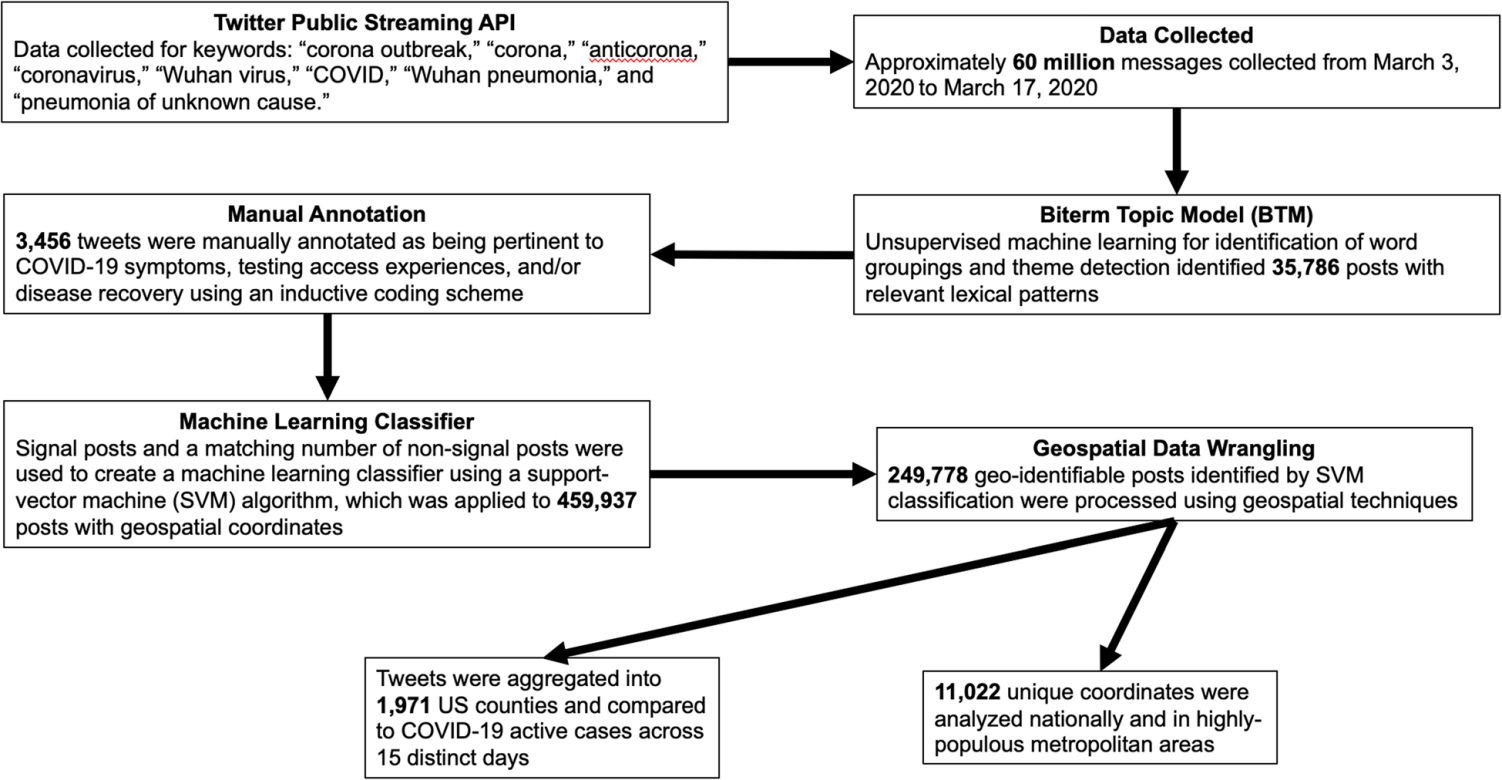
Flowchart describing data collection, processing, and analysis phases for social media posts related to COVID-19

Study methods included interdisciplinary approaches in data mining, mathematical transformations, regression analysis, geospatial statistics, and machine learning for content classification. The distributions predominantly under scrutiny were spatial, though temporal fluctuations were also assessed. Data collection and mining was conducted using the computer programming language Python and data analysis was conducted in ArcGIS version 10.6 and R version 3.6.0. Figure 1 provides an overview of the data collection, processing, and analysis phases used in this study.

### Data collection

Data access via the Twitter public API stream was used to prospectively download publicly available posts located in the United States between March 3rd and March 17th, 2020, inclusive. Data collection commenced in early March and was terminated for the purposes of this study when data were sufficient to allow for testing the longitudinal prediction of cases from social media posts with a two-week time-lag. This timeframe can also be characterized as occurring towards the end of the early outbreak period in the United States, as on March 13th, former President Donald Trump declared COVID-19 a national emergency, an event emphasizing the seriousness of the outbreak and unlocking billions in federal funds to fight its spread.

Keywords used to obtain tweets from the public API stream were intended to encompass a broad representation of conversations regarding COVID-19 at the time. These were “corona outbreak,” “corona,” “anticorona,” “coronavirus,” “Wuhan virus,” “COVID,” “Wuhan pneumonia,” and “pneumonia of unknown cause.” these keywords were chosen on the basis of structured manual searches conducted on twitter that detected content related to the COVID-19 outbreak as posted by users, and they have also been validated as being able to identify tweets pertaining to COVID-19 conversations in prior studies [29, 30]. Approximately 60 million messages were collected during this timeframe. Prior studies suggest that collecting data from the twitter public streaming API will generate a random sample of approximately 1% of all public tweets having these keywords, though selection of keywords, volume of overall tweets, and other factors may impact this approximation (see “limitations” section) [31]. Of these tweets, 459,937 had available geospatial information in the metadata of collected messages.

Geospatial information was in the form of latitude and longitude coordinates. The original source of this information was information generated from the user’s device where Twitter users are required to opt in to enable geolocation. For the purposes of this study Twitter IDs were removed from datasets prior to analysis to ensure appropriate de-identification. Posts with these coordinate data are made available to third parties via the Twitter API. There are a number of reasons why twitter users may choose to geolocate or geotag their tweet. Reasons for lack of geolocation include changes in Twitter’s privacy policy and individual privacy concerns about sharing data and not opting-in to geolocation services/apps. Specifically, in 2015 Twitter changed its terms to require explicit op-in to share precise location data (i.e. GPS data) whereas previously precise data was included when geotagging tweets. Reasons why users may geolocate include a preference to geotag their location when tweeting and integration or use of other applications.  COVID-19 cases at the U.S. county and national levels were available from the 2019 Novel Coronavirus COVID-19 (2019-nCoV) Data Repository, actively maintained on GitHub by the Johns Hopkins University Center for Systems Science and Engineering, which collects case information reported from a variety of validated sources [32]. Cases were obtained for each day when posts were collected on Twitter. Active case counts were used in regression modeling, computed by subtracting county-level recoveries and deaths from confirmed cases. Normalization of tweets for national and local analysis was done by dividing the number of tweets by the amount of people living in a given county or census tract. Population at these ecological units was available from the US Census Bureau.

### Content analysis using machine learning

In a prior published study, we used an unsupervised machine learning approach, called the biterm topic model (BTM), to identify users self-reporting COVID-19 symptom related experiences and concerns (e.g., lack of access to testing when having symptoms) from the 60 million-tweet corpus collected during the study period. Coding involved five thematic categories/codes for identification of “signal” tweets (i.e., tweets that were confirmed as associated with self-reporting of symptoms and concerns after manual annotation). Results from the study reported high inter-rater reliability (κ = 0.98); with a more detailed description of these methods available in Mackey et al., 2020 [29]*.* In brief, 35,786 posts were clustered by BTM and identified as containing highly correlated word patterns thought to be associated with symptom-related conversations, with 3465 of these posts then positively identified via manual annotation as associated with self-reporting of symptoms and concerns.

These signal posts largely did not contain geospatial information but were nevertheless detected during the early outbreak period and were used for this study. This initial set of training data, along with a matching number of posts coded for non-signal (i.e., posts that were false positives and determined by manual annotation to not be related to self-reporting of symptoms), were used for further supervised machine learning classification tasks using a support-vector machine (SVM) algorithm. The SVM classifier was applied to the 459,937 posts in this study with geospatial coordinates to identify 249,778 posts whose text content is more consistent with self-reported user Twitter messages related to COVID-19 symptoms/concerns, therefore excluding “noise” associated with tweets that were about news coverage, satire, and other topics not related to symptoms and concerns. This final labeled subset of 249,778 geo-identifiable posts was used in all regression models computed in this study.

As our training set explicitly excluded posts that appeared to originate from bots in its signal dataset (including signal data we observing observed generally included longer interactions with other users, original content, and profile information that had individually identifiable information or biographies), the output of the SVM classifier is likely to exclude from its classification similar bot-like traffic. In addition, we observed that the average ratio of users’ followers to following was 1607:78, and only 111 users had accounts created recently in 2020 in the prior published study, which are all macro characteristics indicative of non-bot traffic.

### Longitudinal analysis

Analysis of signal tweets specifically located for the United States involved scrutiny of the longitudinal relationship between tweets and cases at the U.S. county level. Bivariate regressions were conducted to investigate the strength of relationships between county-level tweets and county-level active cases. These models were computed to compare the distribution of tweets on the same day as the distribution of active cases, as well as for every combination of time-lagged tweets with active cases. R^2^ values were used to assess the fit of linear relationships and were compared to Nagelkerke’s R^2^ to assess the fit of exponential relationships for the set of same-day or time-lagged relationships. Nagelkereke’s method provides a range from zero to one, as with the R^2^ statistic for linear relationships, in computing a fit relative to a nested null model without predictors.

### Geospatial analysis

In order to explore the county-level distribution of all geolocated tweets with COVID-19 keywords within the United States, tweets were aggregated across the March 3rd - 17th data collection period and divided by county-level population based on data from the US Census Bureau. Geospatial cluster analysis was conducted for the top five most populous cities in the United States. For cities bounded by the perimeter of a single county, the distribution across census tracts was analyzed at the county level; otherwise, the distribution across census tracts was analyzed at the city level. This strategy was undertaken in order to relay relevant distributions across space, particularly with cities contiguous with numerous other cities and towns. Unique coordinates from the overall study period were utilized for cluster analysis of all five areas, so as to prevent clustering statistics from being biased toward locations of individuals with higher volumes of social media posting. For areas with sufficient sample size, additional analysis was done on the first day (March 3rd) and the last day (March 17th) of the study period, in order to relay change in the distribution of social media messages. The computational analysis itself involved calculation of the Getis Ord Gi* statistic for each census tract within the area. These *G* statistics were used to obtain corresponding *z* scores, which were then visualized to relay high-value “hot” spots and low-value “cold” spots.

## Results

A total of 459,937 tweets included geospatial information and originated from 11,022 unique coordinates. Tweets were available for 1971 US counties (55% of US counties), with tweets per capita ranging from 0.0000076 per capita to 0.75 per capita. National analysis assessed active cases from March 3rd, which totaled 59 cases across 24 counties ranging from 1 to 14 cases per county; increasing on March 17th to 5911 active cases (representing close to a 10,000% increase) across 523 counties ranging from 1 to 807 per county.

### Longitudinal analysis

Regression models comparing non-normalized tweets and active cases tended to exhibit better fit as the day of active case data became further from the day of tweet data (Table 1). This prediction was especially strong for exponential models, with the fit of active cases by COVID-related tweets reaching an R^2^ of 0.76 for the exponential model with tweets from March 4th predicting active cases 13 days later. Average R^2^ for same-day prediction was 0.16 for linear models and 0.15 for exponential models. Although only one comparison was available, R^2^ for 14-day prediction was 0.49 for the linear model and 0.73 for the exponential model. The tweet covariate was not statistically significant when predicting cases on March 3rd, March 4th, or March 5th. Conversely, 96% of bivariate models for subsequent days exhibited *p* values under 0.05 for the tweet covariate.

**Table 1:**
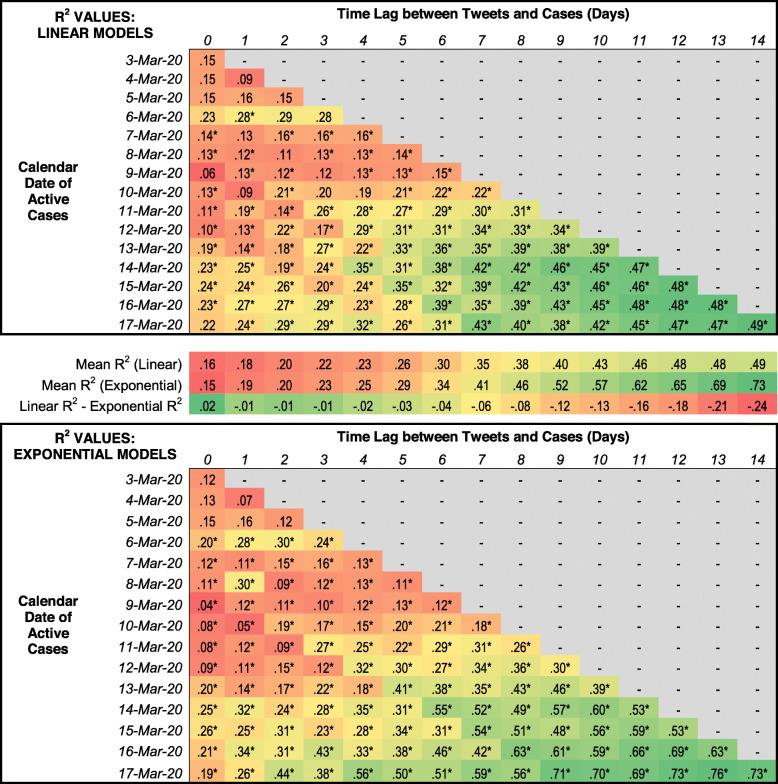
R^2^ values for regression models with number of active COVID-19 cases regressed on number of tweets related to COVID-19 at the county level. Linear and exponential models were computed for both same-day tweets and time-lagged tweet day predicting active cases. Greener shading indicates higher R^2^ values within that table; otherwise, shading is relative to row values

*p value for tweets covariate under 0.05

### Geospatial analysis

Between March 3rd and March 17th, we collected 249,788 posts which were classified as related to COVID-19 user-generated symptoms and concerns as outputted by the SVM classifier. These posts had 11,022 unique geospatial coordinates. To illustrate the difference in social media communication between early March and mid-March, we compared tweets obtained at the beginning of the study period (March 3rd) with those obtained 2 weeks later (March 17th). We detected 3842 (34.9%) posts on March 3rd and 8420 (76.4%) on March 17th. Within the five most populous cities in the United States (or their respective encompassing counties), there were 95 unique coordinates from New York City, with 35 (36.8%) on March 3rd and 66 (69.5%) on March 17th; 178 unique coordinates from Los Angeles County, with 58 (32.3%) on March 3rd and 147 (82.5%) on March 17th; 86 from Cook County (i.e. Chicago), with 27 (31.4%) on March 3rd and 59 (68.6%) on March 17th; 81 from Houston, with 26 (32.1%) on March 3rd and 73 (90.1%) on March 17th; and 40 from Maricopa County (i.e. Phoenix) with 13 (32.5%) on March 3rd and 25 (62.5%) on March 17th. Therefore, across all city areas, approximately one-third of the number of locations interacting with the COVID-19 topic were represented in early March. The number of locations approximately doubled by mid-March, consistent with the national trend in increase in number of locations tweeting about COVID-19.

**Fig. 2:**
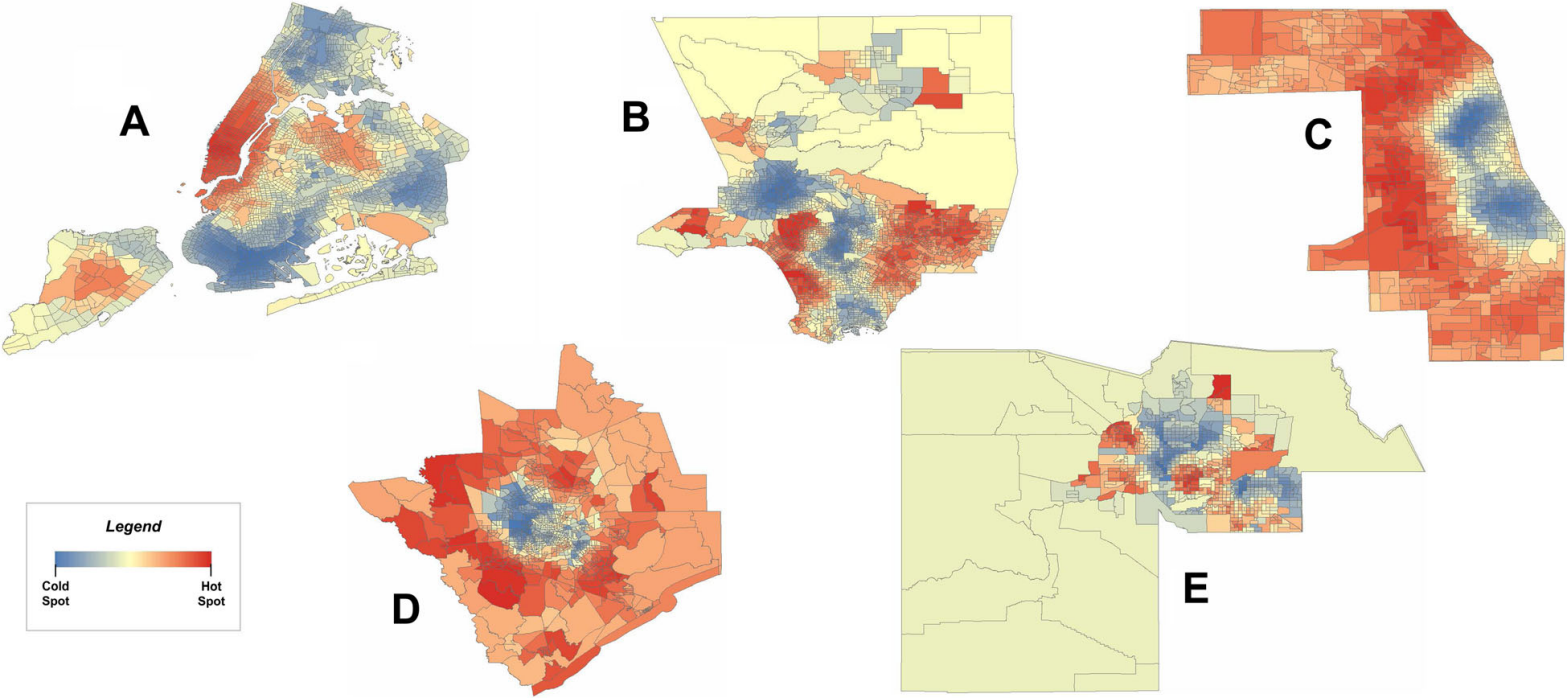
*Z*-scores for the Getis Ord Gi* statistic, indicating geospatial clustering of tweets about COVID-19 from (**a**) New York City, (**b**) Los Angeles County, (**c**) Cook County (i.e. Illinois), (**d**) Houston, and (**e**) Maricopa County (i.e. Phoenix)

Geospatial cluster analysis was conducted for each major metropolitan city area. In New York City, a cluster of tweets was detected in Manhattan, which is the most densely populated area of the city. Conversely, in Los Angeles County, the densely populated downtown area was labelled as a cold spot, whereas the relatively residential areas of West Los Angeles and San Gabriel Valley were labelled as hot spots. The same pattern was observed for Cook County (i.e., Chicago) and Houston, where city centers exhibited lower levels of social media conversations related to COVID-19 symptoms and concerns. However, the distribution of tweets within Maricopa County (i.e., Phoenix) seemed more consistent with that of New York City, with relatively high representation of COVID-19 conversations in the densely populated city center (Fig. 2). These results may reflect different underlining patterns of twitter user’s engagement or geolocation in different city and residential environments, or more or less engagement on COVID-19-related topics due to specific community considerations (e.g., on March 17th, New York City had 182 new cases and Los Angeles County had 50 new cases), though these patterns require further study and generation of additional hypotheses.

**Fig. 3:**
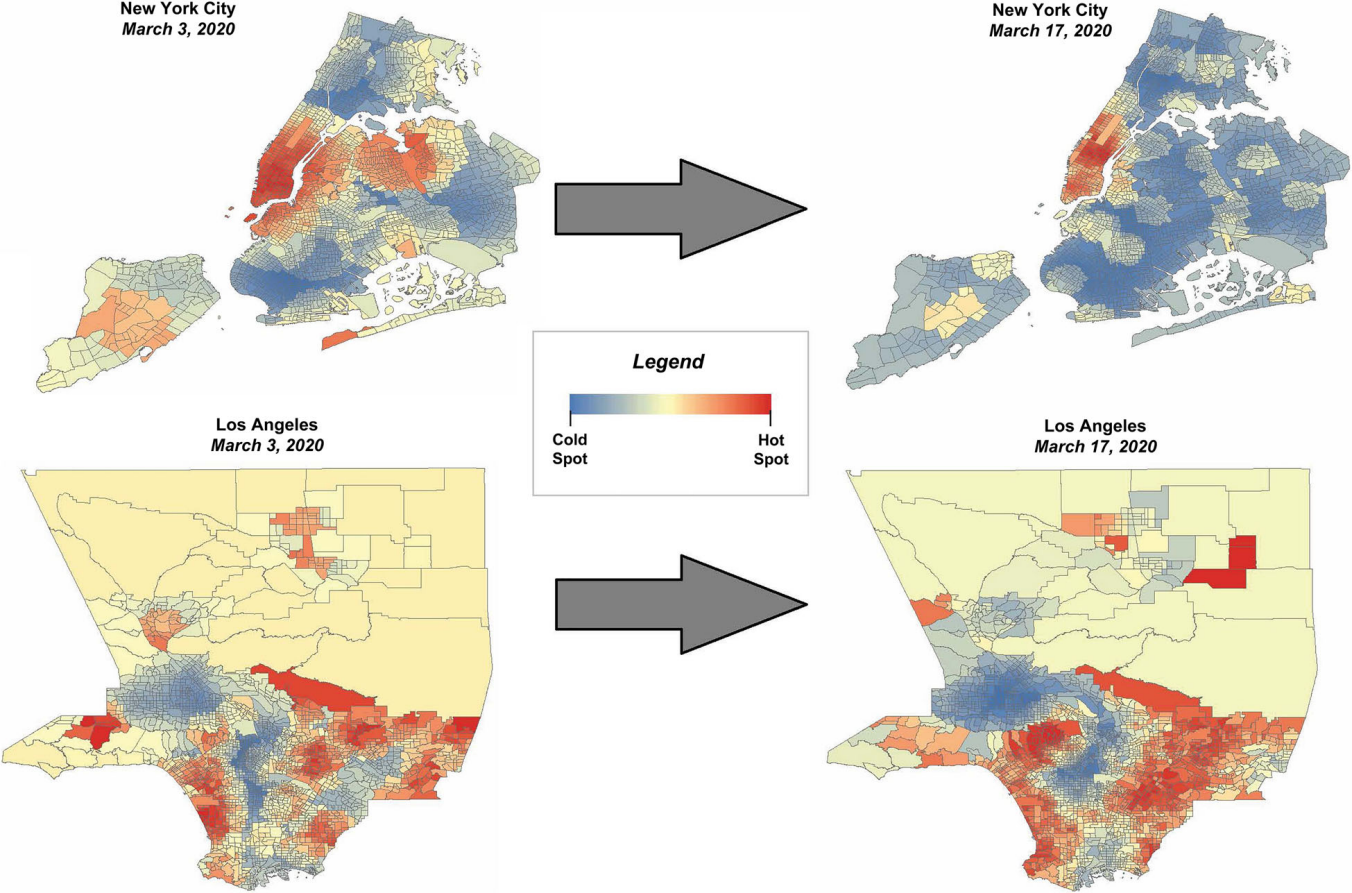
Changes in *z*-scores for the Getis Ord Gi* statistic, relaying clustering at the start of the study period (March 3rd) and the end of the study period (March 17th) for New York City and Los Angeles County

Analysis for different time points was possible for New York City and Los Angeles County. On March 3rd, the distribution of coordinates in New York City spanned across lower and central Manhattan, radiating across the East River into parts of Brooklyn and Queens. Despite more social media activity on March 17th, the distribution of coordinates in New York City became much more concentrated within the island of Manhattan. The opposite trend was observed in Los Angeles County. On March 3rd, small clusters of tweets were detected from Los Angeles International Airport and some areas of San Gabriel Valley. On March 17th, these small clusters appeared to have grown to encompass most areas within the county’s South Bay, Westside, San Gabriel Valley, and Southeast regions (Fig. 3).

## Discussion

From an initial dataset of 60 million tweets, this study used a combination of geospatial analysis, statistical testing, and machine learning to analyze approximately 460 k tweets that contained COVID-19-related keywords that were geolocated in just over half of all counties in the United States during the early stages of the pandemic. We observed variation in clustering of tweets within populous metropolitan areas, indicating sub-regional differences in patterns of social media communication about COVID-19.

Results of this study are primarily exploratory and are important in generating further hypotheses to better characterize social media communication dynamics at early stages of a public health emergency, particularly in the context of a novel emerging infectious disease and when there is a lack of accurate information on case counts, as this period was marked by underreporting of active and asymptomatic cases due to lack of testing capacity [12]. Overall, this study revealed a number of quantitative aberrations, discrepancies, and findings that require further study that could help in better assessing the epidemiological and communication characteristics of the ongoing COVID-19 pandemic using geolocated tweets as a proxy indicator for community attention to disease outbreaks, with possible insights related to disease transmission trends over time.

Analysis of county-level data in the United States also suggested a time lag between Twitter posts and predictive power for officially reported COVID-19 cases. In this study, posts exhibited much better fit for reported active cases as the time gap between Twitter posts and cases increased, especially in exponential models. The reason for this discrepancy may be that social media users are responding to the perceived risk of COVID-19 due to news coverage and conversations generated from other users’ experiences earlier than official reporting of cases by state public health agencies [33]. Specifically, there is generally a lag with public health surveillance reporting, which can also be impacted by the availability and speed of laboratory testing [34]. Hence, this study’s approach can potentially provide closer to real-time insights by identifying communities where self-reporting of symptoms and public concern about disease transmission are experiencing statistical and geospatially specific fluctuations.

Analysis of the areas for the five most populated cities in the United States revealed some consistencies and also some differences in relation to Twitter engagement on COVID-19-related topics. All cities appeared to follow the national longitudinal trend of increasing tweets and corresponding case counts, where approximately one-third of all cities analyzed included Twitter discussions related to COVID-19 on March 3rd, which then doubled by March 17th. This result appears to indicate that, in addition to the total number of Twitter posts following a similar longitudinal trend as local COVID-19 rates, the number of unique locations interacting with this issue was also indicative of the evolving national concern about the pandemic.

Specifically, temporal differences were observed between New York City and Los Angeles County, where clustering of highly tweeted areas became more concentrated in New York City and less concentrated and dispersed in Los Angeles County. As New York City exhibited extremely high COVID-19 rates in mid-March compared to the rest of the country, this result may partly be due to highly concentrated user engagement in specific areas of the city, mainly Manhattan (with users in this area possibly more likely to geotag their tweets), actively discussing COVID-19 topics. Los Angeles County exhibited the opposite trend, where the distribution of tweets became more dispersed between March 3rd and March 17th, possibly reflecting the fact that case counts were relatively lower in this area compared to other states/cities. Furthermore, lack of highly concentrated tweet geographic clusters may indicate that public concern about the outbreak was still being formulated, particularly as case counts continued to increase locally. In both city-specific groups of tweets, we observed that users commented about concerning issues related to an absence of people/crowds, decrease in traffic, and store/event closures, towards the end of the study period. This may reflect growing awareness to the pandemic’s severity as the pandemic progressed.

Overall, the cities of New York and Phoenix exhibited different Twitter clustering patterns than those for the areas of Los Angeles, Chicago, and Houston. In New York and Phoenix, clusters were generally observed from relatively densely populated city centers. However, in Los Angeles, Chicago, and Houston, clusters were mostly observed outside of city centers. Further research is needed to assess why these city and residential specific variations may be occurring, which could be influenced by differences in dynamics between working and living in different cities (including access to public transportation, commuting for work, and the impact of stay-at-home orders on working and living conditions specific to cities or states) [35]. Areas that exhibited similar tweet clustering (e.g., the Phoenix area and New York City) require further study to assess if there are similar patterns of user risk-perception or COVID-19-related self-reported behavior.

Importantly, our study builds on a growing body of infoveillance literature using geolocated data to explore potential disease transmission and online communication dynamics using publicly available social media data. This includes a 2016 study of Japanese tweets containing influenza symptoms which found a similar time lag between the rate of tweets with forecasting words and the national influenza rate [36]. A separate study of Korean tweets from 2016 found that tweets with keywords related to Middle East respiratory syndrome (MERS) were more predictive of the South Korean quarantine rate as the time lag increased, but less predictive of laboratory-confirmed cases [37]. Finally, a 2010 study of English-language tweets about the H1N1 pandemic found that tweets which were automatically coded as indicative of personal disease experience, based on keywords, exhibited high correlation with personal disease experience after manual verification of tweets [38].

Throughout the COVID-19 crisis, maps and data dashboards have been popularly used to describe the extent and distribution of the pandemic and offer actionable public health insights [32, 39, 40]. However, these maps have primarily focused on the disease distribution itself (e.g., visualizing validated cases, testing and vaccine centers, and requesting users to self-report symptoms), whereas social consequences of the disease (such as those discussed in social media posts) may also provide useful insights warranting the production of map visualizations [41, 42]. Furthermore, there exist powerful geospatial and statistical methods that can applied to these data to assess specific risk factors associated with geolocated communities, including examining potential COVID-19-related challenges such as health disparities, lack of access to testing/treatment, and assessing the impact of policy on pandemic response and human behavior [43]. This form of digital syndromic COVID-19 surveillance can generate previously undiscoverable insights not readily available from other data sources.

Infoveillance-derived metrics, such as the relative frequency of posts from a given area and the change over time of tweets from specific communities, may also be helpful in coordinating public health communication strategies to effectively disseminate targeted information and education relevant to pandemic response, needed public health interventions, and associated clinical care [19, 44]. Geospatially-resolute infoveillance statistics may also be helpful after the initiation of public health communication programs, as they can provide evidence on the comparative effectiveness of strategies and/or the variation in community-level absorptive capacity for a given communication strategy [33, 45]. Furthermore, geospatial variation in COVID-19 risk factors has previously been tied to discrepancies in patterns of hospitalized care for COVID-19 [46], and spatial concentrations of healthcare workforce personnel have been associated with COVID-19 case distributions [47, 48]. Concordantly, further research should assess how geospatial variation in online communication dynamics of COVID-19 may be predictive of patterns in patient care and population-based healthcare metrics.

### Limitations

Findings from this study are subject to a number of key limitations. Importantly, only a fraction of all tweets are geolocated, which raises the possibility of sampling bias with respect to the overall tweet corpus. Aggregated analyses correspond to 2 weeks of social media communication and therefore may have limited generalizability beyond the early outbreak period. Furthermore, some communities and their specific demographic features may have a greater propensity to post Twitter messages or geolocated tweets, regardless of conditions experienced in any of its users’ communities. While it is possible that this error is approximately systemic, and thereby may not appreciably contribute to the discovery of spurious relationships, little analysis has been done to verify whether the proportion of posts responding to local conditions is consistent across geospatial units. Similarly, we have considered the variation in COVID-19 cases to be reflective of true variation at an artificially deflated magnitude, due to insufficient testing. However, testing capacity of local public health bodies may have appreciably varied during the study period, potentially resulting in erroneous variation, in addition to the suspected erroneous variation in magnitude. This study is intended to be primarily hypothesis generating, and findings from this study should be further validated in more highly controlled settings while also leveraging additional sources of both structured and unstructured data. For example, a study in a manageable set of smaller communities should seek to determine whether variation in social media data is highly predictive of community caseloads that were obtained by communities with similar levels of testing at this early stage of the pandemic, and also attempt to account for variations that may relate to public health policy decisions at the local level. Such future studies may also seek to assess differences in the predictive power of social media messages at different intervals from the caseload prediction time point.

## Conclusion

Results from this study suggest that social media communication dynamics during the early stages of a global pandemic exhibit a number of geospatial specific variations and that engagement of these topics may be predictive of future confirmed case counts, though further studies to validate these findings are needed. Across five major US cities, geospatial patterns of tweets about pandemic-related experiences and concerns revealed variations in geospatial hot and cold spots of tweet locations between metropolitan communities, with suggested further variations relating to how these clustering patterns change over time. The utility of social media data as an infoveilance data layer for measuring early concern about infectious disease outbreaks warrants further study, as does the potential moderating effect of concern on behavior-related prevention of transmission.

## Data Availability

The datasets used and/or analyzed during the current study are available from the corresponding author on reasonable request.
